# Influence of Time-of-Day on Maximal Exercise Capacity Is Related to Daily Thermal Balance but Not to Induced Neuronal Activity in Rats

**DOI:** 10.3389/fphys.2016.00464

**Published:** 2016-10-14

**Authors:** Frederico S. M. Machado, Daniela R. C. Fóscolo, Maristela O. Poletini, Cândido C. Coimbra

**Affiliations:** Department of Physiology and Biophysics, Institute of Biological Sciences, Universidade Federal de Minas GeraisBelo Horizonte, Brazil

**Keywords:** forced exercise, fatigue, thermoregulation, circadian, photoperiod, spontaneous locomotor activity, neural activation

## Abstract

In the present study, we investigated whether the daily fluctuations of internal body temperature (T_b_) and spontaneous locomotor activity (SLA) interact with the thermal and neuronal adjustments induced by high-intensity aerobic exercise until fatigue. The body temperature and SLA of adult Wistar rats (*n* = 23) were continuously recorded by telemetry for 48 h. Then, the rats were subjected to a protocol of graded exercise until fatigue or rest on the treadmill during light and dark-phases. T_b_, tail skin temperature and ambient temperature during each experimental session were recorded. At the end of the last experimental session, the animals were anaesthetized; the brains were perfused and removed for immunohistochemical analysis of c-fos neuronal activation. The daily rhythms of SLA and T_b_ were strongly correlated (*r* = 0.88 and *p* < 0.001), and this was followed by a daily oscillation in both the ratio and the correlation index between these variables (*p* < 0.001). Exercise capacity was associated with a lower resting T_b_ (*p* < 0.01) and was higher in the light-phase (*p* < 0.001), resulting in an increased capacity to accumulate heat during exercise (*p* < 0.01). Independent of time-of-day, high intensity exercise strongly activated the hypothalamic paraventricular nucleus (PVN), the supra-optic nucleus (SON) and the *locus coeruleus* (LC) (*p* < 0.001) but not the suprachiasmatic nucleus (SCN). Taken together, our results points toward a role of the circadian system in a basal activity control of the thermoregulatory system as an important component for the onset of physical activities. In fact, rather than directly limiting the adjustments induced by exercise the present study brings new evidence that the effect of time-of-day on exercise performance occurs at the threshold level for each thermoregulatory system effector activity. This assumption is based on the observed resilience of the central clock to high-intensity exercise and the similarities in exercise-induced neuronal activation in the PVN, SON, and LC.

## Introduction

Thermal balance is a key feature of *homeostasis* maintenance and defense in homoeothermic animals. In the case of an abrupt and persistent internal imbalance such as prolonged running exercise, *homeostasis* is challenged, demanding a coordinated, and integrated response of the central nervous system (CNS) to reach a new safe steady state until exercise is interrupted. We have previously shown that time-of-day plays an important role in both spontaneous and forced physical activity capacity through changes in the initial thermal state (Machado et al., 2015). However, the central mechanisms that might explain these influences remain to be further elucidated.

Internal body temperature is one of many factors that determine fatigue and exercise capacity (Cheung and Sleivert, 2004). Exercise increases muscular and cardiovascular activity resulting in higher metabolic rate an, consequently, in greater heat production (Webb, 1995; Pires et al., 2007; Rodrigues et al., 2008). The CNS plays a fundamental role in the coordination and integration between the distinct adjustments prompted by exercise in order to maintain homeostasis. Brain regions implicated in autonomic response, such as the PVN, SON, and LC (Pacák and Palkovits, 2001; Mastorakos et al., 2005) are activated during high-intensity exercise (Soya et al., 2007; Yanagita et al., 2007; Barna et al., 2012). In addition, both the PVN and SON, nuclei activated in an intensity-dependent manner during exercise, have been implicated in ACTH release, lactate accumulation, increased glycaemia, and adrenaline secretion induced by exercise (Soya et al., 2007; Lima et al., 2014a). Activation of these nuclei also might be involved in tail vasodilation during exercise (Nagashima et al., 2000; Lima et al., 2014a).

The suprachiasmatic nucleus (SCN) has been known as the “central clock” that coordinates most of circadian subsystems distributed through the CNS and the periphery (Pittendrigh, 1993; Mendoza and Challet, 2009; Bass and Takahashi, 2010; Albrecht, 2012). Lesions of the SCN results in the loss of regulation of circadian locomotor activity, body temperature and heart rate (Satinoff et al., 1993; Li and Satinoff, 1995; Scheer et al., 2005). Interestingly, SCN transplants to SCN-ablated animals restored these rhythms, thus confirming its importance as an orchestrator of the internal clock (Lehman et al., 1987). Direct and indirect projections of the SCN to other brain regions related to autonomic and behavioral responses are thought to synchronize most of the physiological systems according to the light/dark and inactivity/activity cycles (Kriegsfeld and Silver, 2006).

The coupling between the inactivity/activity and light/dark cycles results in a circadian cycle of thermal balance. During the active phase, individuals increase their physical activity and food intake, leading to a peak in body temperature that is tightly associated with these behavioral patterns (Büttner and Wollnik, 1982; Kotz et al., 2008). In fact, the degree of influence between locomotor activity and body temperature changes throughout the day (Weinert and Waterhouse, 1998; Refinetti, 2003; Machado et al., 2015), possibly through the activity of the CNS, suggesting a rhythm underlying the internal set point of the thermoregulatory system that may have implications in thermoregulation during exercise performed at different times of the day.

Exercise increases heat production mainly through contraction-induced ATP hydrolysis as a function of activity workload (Webb, 1995). To counterbalance heat production and storage during the onset of exercise, the thermoregulatory system increases vasodilation tone to the skin surface leading to improved heat loss. The transient delay between heat production and skin vasodilation results in heat storage and consequently, in increased body temperature. Reaching critical levels of body temperature (Fuller et al., 1998; Walters et al., 2000) or higher rates of heat storage (González-Alonso et al., 1999; Cheung and Sleivert, 2004; Lacerda et al., 2005; Lambert et al., 2005) may impair running capacity and lead to exercise interruption. This process is strictly regulated by the CNS, possibly through the pre-optic area (POA), the anterior and posterior hypothalamic nuclei involved in the thermal and stressor response, and the brainstem (Hasegawa et al., 2005; Meeusen et al., 2007).

The changes in thermal balance during low-to-moderate intensity treadmill running exercise are influenced by the time-of-day (Tanaka et al., 1990). Furthermore, environmental light can affect thermoregulation during rest and exercise conditions during both the light and dark-phases of the rest/activity cycle (Hasegawa et al., 2000). Thus, provided that time-of-day changes in initial body temperature affects physical performance (Machado et al., 2015), it could be possible that the activity of the central clock plays a role in modulating the activity of the thermoregulatory system and consequently affects exercise performance. Hence, the present work aimed to investigate whether changes in exercise capacity and spontaneous locomotor activity related to time-of-day cyclic oscillations in body temperature could be associated with CNS neuronal activation induced by high-intensity exercise.

## Methods

### Ethical statement

All of the described experimental procedures were approved by the local ethics committee (CETEA/UFMG) under the protocol number 139/2008 and followed the guidelines for laboratory animal handling and experimentation in line with the European Convention for the Protection of Vertebrate Animals used for Experimental and other Scientific Purposes (1985).

### Animals

A total of 35 adult male Wistar rats were used during this study. Animals were housed in a room with a cycle of 10 h of exposure to dark and 14 h to light, with the lights being turned on at 06:00 (defined as *zeitgeber* time 0, ZT0) and turned off at 20:00 (ZT14). Housing conditions included free access to water and chow (NUVILAB-CRI, PR, Brazil) and a constant ambient temperature of 23°C.

### Surgical procedures

Three days before the first experimental day (described in the next session), animals were anesthetized with an intraperitoneal injection of a solution containing a mixture of ketamine (11.6 mg of 10% ketamine per 100 g of animal body weight) and xylazine (0.57 mg of 2% xylazine per 100 g of animal body weight). A ventral incision at the *linea alba* was made to introduce a telemetric sensor (G2 E-Mitter, Mini-Mitter Company, Sun River, OR, USA) into the peritoneal cavity. Each probe was then sutured to the inner musculature before the incision was closed. This procedure allowed the continuous monitoring of both T_b_ and SLA with decreased risk of the internal displacement of the sensor, which could lead to misleading readings due to its position. At the end of the surgery, a single dose of *intramuscular* 24,000 U kg^−1^ procaine penicillin (Pentabiótico Veterinário®, Fort Dodge Animal Health Ltd.) and *subcutaneous* 1.1 mg kg^−1^ non-steroidal anti-inflammatory analgesic (Banamine®, Scering-Plough, SA) were administered.

### Recordings of the daily cycle of T_b_ and SLA

On the first day of the experiment, animals that participated in exercise to fatigue and the resting group were placed inside individual cages situated over a telemetry signal receiver. Over the course of 48 h, they were kept under these conditions to evaluate the daily oscillation of SLA and T_b_.

Each animal was individually housed inside standard cages in a calm and separate room with the photoperiod set at 14 h of artificial light (lights on at 06:00) followed by 10 h of darkness (lights off at 20:00) and a controlled ambient temperature (23°C). Water and food (standard rat chow, NUVILAB) were provided *ad libitum*. In order to avoid an influence of handling by the experimenter, only the data generated 12 h after the initiation of recording was analyzed.

Cages were positioned over a telemetry signal receiver (ER-4000 Energizer/Receiver, Mini-Mitter Company, Sun River, OR, USA), which was previously configured to record the specific signal frequency emitted by the sensor probe that was previously implanted into the peritoneal cavity. Data were transmitted and stored on a computer installed with VitalView Software (VitalView® Data Acquisition System Software v. 4.0, Mini-Mitter Company, Sun River, OR, USA). T_b_ (°C) (accuracy of ±0.1°C) and SLA (arbitrary units min^−1^) were continuously recorded every minute for 48 h. Locomotor activity, or gross motor activity, represents the movement of the E-mitter in the longitudinal, horizontal and transversal axis over the receptor plate during each bin. The means for each phase of the photoperiod were calculated for comparison and to confirm that the experimental setup was in agreement with the nocturnal habits described for Wistar rats. Furthermore, these averages were also used to study the correlation between these two parameters throughout the day and with exercise capacity.

### Treadmill protocols

To verify whether physical performance is influenced by the light/dark cycle and that this difference is influenced by thermal balance oscillations, exercise capacity, internal body temperature (T_b_) and tail skin temperature (T_sk_) at daytime and night-time were compared during rest and exercise until fatigue.

On the first day following the previous experiment, animals from both experimental groups were acclimated to a motor-driven treadmill adapted for small rodents (TREADMILL LE 8706, Letica® Scientific Instruments, Barcelona, Spain). This familiarization process took 5 days and consisted of running for 5 min at a speed ranging from 10 to 15 m min^−1^ with a constant slope of 5°. A thermocouple (series 409-B, Yellow Springs Instruments, Dayton, OH, EUA) was taped to the lateral surface of the skin, 1 cm from the base of the tail to measure T_sk_ and to acclimate the animals to the procedure. A slight electrical stimulation (0.4 mA) was provided to insure that the animals started to exercise.

Animals were randomly assigned to the rest (*n* = 12) or exercise to fatigue (*n* = 11) groups. The experiments were performed during the early moments of the light and dark-phases of the luminosity cycle in a room with a constant ambient temperature of 24 ± 1°C. Light-phase exercise started at the moment when the lights were turned on (at 06:00 or ZT0), and dark-phase exercise started when the lights were turned off (at 20:00 or ZT14). During the night phase protocols, environmental red light was provided to the experimental room to avoid white light effects on thermoregulatory adjustments during rest and exercise (Hasegawa et al., 2000). Each animal underwent two experimental sessions during both the day and the night phase.

To minimize the acute effects of environmental changes of the measured variables, 2 h before the phase change, animals were weighed and placed in the same room of the experiments. One hour before the phase change, they were positioned inside the treadmill and T_b_ was recorded every 10 s until the end of the experiment. Immediately after the phase change, the thermocouple was taped to the lateral surface of the tail and connected to a precision thermometer (4600 Precision Thermometer, Yellow Springs Instruments, OH, EUA). In sequence, exercise, or registry for resting conditions was initiated.

The graded intensity exercise protocol consisted of running at an initial speed of 10 m min^−1^ and increments of 1 m min^−1^ every 3 min until fatigue. Fatigue was set as the moment when rats could not keep pace with the treadmill, which was defined as time to fatigue (TF) (Balthazar et al., 2009; Prímola-Gomes et al., 2009). After that moment, animals remained inside the treadmill with the thermocouple attached for 30 min for recovery monitoring. The resting protocol consisted of staying on the treadmill for 90 min. During the second experimental session 90 min after fatigue (Lima et al., 2014b; Santiago et al., 2016; for a short review of c-fos protein expression, please refer to Kovàcs, 1998), animals were deeply anesthetized with ketamine and xylazine and were transcardially perfused with 40 mL of heparinized 0.01 M phosphate-buffered saline (PBS), followed by 400 mL of 4% paraformaldehyde (PFA) in 0.2 M phosphate-buffered saline (pH 7.4). The brains were removed for subsequent immunohistochemical analysis.

### Immunohistochemistry

After perfusion, the brains were removed and post-fixed in 4% PFA for 2 h at 4°C. Thereafter, the brains were stored in cold 30% sucrose for 72 h (until the brains sank) and frozen in isopentane 99% (C_5_H_12_) at −50°C. The brains were then stored at −80°C until sectioning. Coronal sections of 40 μm of the forebrain including the SCN, paraventricular nucleus (PVN) and supra-optic nucleus (SON), as well as thinner 35 μm slices of the *locus coeruleus* (LC) at the brainstem (Paxinos and Watson, 2005), were cut with a cryostat and stored in cryoprotective solution at −20°C until the immunohistochemistry assay. Tissues were initially washed with 0.01 M phosphate-buffered saline (PBS). Unless otherwise mentioned, all solutions were prepared in PBS, and sections were washed with PBS between all steps. The sections were sequentially incubated with 0.1 M glycine, 1% hydrogen peroxide, 0.4% Triton X-100 and 2% bovine serum albumin (BSA). The sections were incubated in a primary antibody directed against c-Fos (Ab5 rabbit polyclonal antibody; Calbiochem, EMD Chemicals, Gibbstown, NJ) at 1:10,000 in PBS containing 0.3% Triton X-100 and 1% BSA (all antibodies were diluted in the same solution) for 40 h at 4°C, then with a biotinylated universal horse IgG (Vectastain Elite ABC kit, Vector Laboratories, Burlingame, CA, USA) at 1:600 for 2 h at room temperature, and finally with avidin-biotinylated horseradish peroxidase complex (Vectastain Elite ABC kit, Vector Laboratories) at 1:400 for 1 h. Sections were stained with a solution of nickel sulfate (25 mg mL^−1^), 3,3-diaminobenzidine-HCl (DAB) (0.2 mg mL^−1^), and 0.03% H_2_O_2_ in 0.175 M acetate-buffer. Subsequently, the sections were mounted onto gelatine-coated slides, air-dried, rinsed in a graded ethanol series, cleared in xylene and coverslipped with Entellan (Merck).

The number of c-Fos-immunoreactive (ir) cells was counted (ImageJ) in images taken with a digital camera (Evolution LC Colour, Media Cybernetics, Silver Spring, MD, USA) attached to a light microscope (Olympus BX-50, Tokyo, Japan) by two experimenters blinded to the experimental groups. For a cell to be considered c-Fos-ir, the nucleus was required to be stained black. The number of c-fos-ir neurons was quantified bilaterally in the SCN (between −0.48 and −0.96 from the bregma), PVN (between −1.72 and −1.92 mm from the bregma), SON (between −0.96 and −1.20 mm from the bregma) and LC (between −9.48 and −9–96 mm from the bregma) according to the rat brain atlas (Paxinos and Watson, 2005).

### Calculations

*Workload* (kgm) was calculated as [body weight (kg)] × [(TF (min)] × [speed (m min^−1^)].

*Heat Storage* (cal), the heat accumulated during exercise, was calculated as [ΔT_b_ (°C)] × [m (g)] × [*c*], where m represents body mass in grams, and *c* represents the specific heat of body tissues (0.826 cal g °C^−1^). The *heat storage rate* (cal min^−1^) was determined as [heat storage (cal)] × [TF (min)]^−1^.

The *Heat Dissipation Index* (HDI) was calculated as (T_skin_ − T_amb_) × (T_b_ − T_amb_)^−1^, where T_amb_ represents the corresponding ambient temperature inside the treadmill.

### Statistical analysis

Results were expressed as the mean ± SEM, unless otherwise stated. Statistical significance was set at *p* < 0.05. Changes in T_b_ and SLA during the light/dark cycle were analyzed by *one-way* ANOVA followed by the appropriate *post hoc* test. Comparisons between light and dark-phase parameters were tested using paired Student's *t* test. The association between T_b_ and SLA through 48 h was analyzed by both *one-way* ANOVA of the ratios and by calculating the linear regression slopes at each hour. This analysis was based on our previous finding showing a close relationship during the light-dark cycle (Machado et al., 2015). The effects of time-of-day and exercise on thermal balance (T_b_, T_sk_) and neuronal activation were analyzed by *two-way* ANOVA followed by a *post hoc* test. Differences between exercise capacity, body heat rate and heat storage were verified by paired Student's *t* test. To evaluate the association between variables, Pearson's correlation was used. In order to evaluate thermoregulatory effector activity, a nonlinear four-parameter analysis was employed. This analysis allows estimating the threshold for vasodilation and the sensitivity of the system to the changes in body temperature. The threshold was calculated as the crossing points of the slope lines at the EC50 point of the curve. On the other hand, the ascending slope of the curve determined the system sensitivity.

## Results

### Daily oscillations in T_b_ and SLA

**Figure 1:**
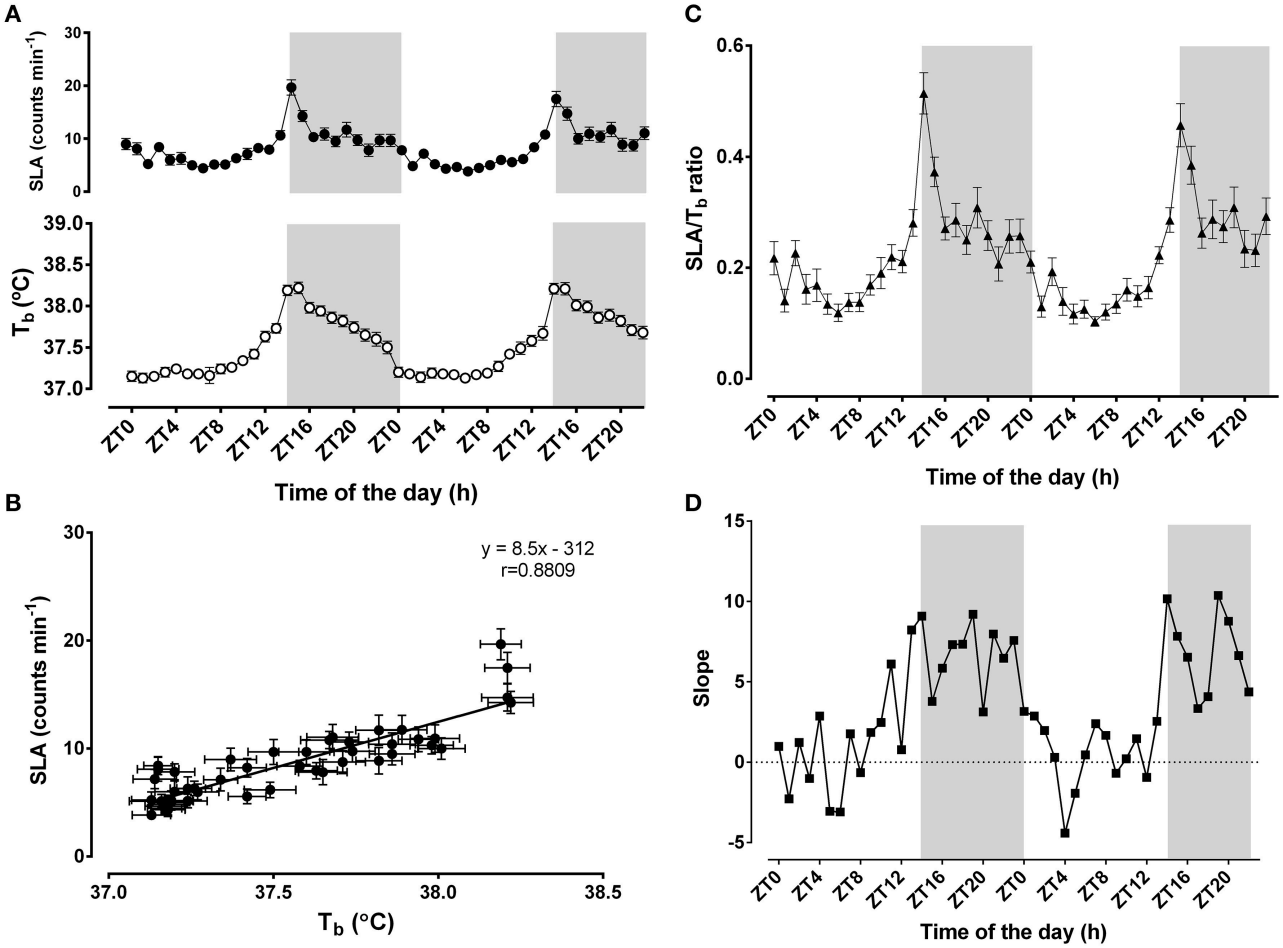
**Daily changes in body temperature (T_b_)and locomotor activity (SLA)**. In **(A)** (*top panel*), black circles correspond to 1-h moving averages of SLA (counts min^−1^), and white circles (*bottom panel)* represent T_b_ (°C). Light/Dark cycles were set at 14/10; the light-phase started at 06:00 (ZT0) when the lights were turned on; the dark-phase (shaded gray background) started at 20:00 (ZT14). In **(B)**, the associations between the daily changes in body temperature and SLA were represented as the correlation between the 24 h-averages of body temperature and SLA. The linear correlation equation and the representative line were added to the graphic. Pearson's *r* coefficient was 0.8809 and *p* < 0.0001. In **(C,D)**, the ratios between SLA and T_b_ (black triangles) and the slopes of the linear regressions at each hour (black squares) were plotted as a function of time-of-day. The results are expressed as the mean ± SEM, except for the slope panel (*n* = 23).

Time-of-day strongly influenced T_b_ and SLA (*F* = 12.26, *p* < 0.0001 for T_b_; *F* = 28.37, *p* < 0.0001 for SLA, *n* = 23) (Figure 1A). Mean T_b_ during 48 h was 37.5 ± 0.05°C, with a peak at ZT15 during the night phase and the nadir at ZT1 during the light-phase. The mean T_b_ during the dark-phase (37.9 ± 0.06°C) was higher than during the light-phase (37.3 ± 0.04°C; *p* < 0.0001). Similarly, the mean SLA was 8.5 ± 0.5 counts min^−1^ over 48 h, peaking at ZT13/14 and reaching its lowest level at ZT0/1. The average SLA during the dark-phase (11.4 ± 0.8 counts min^−1^) was also higher than during the light-phase (6.4 ± 0.3 counts min^−1^; *p* < 0.0001). Overall, these two variables were strongly correlated over the 48 h of monitoring (*r* = 0.88; *p* < 0.0001) (Figure 1B).

To further evaluate the difference in the dark-phase component of the oscillatory pattern of thermal balance, we assessed how the association between SLA and T_b_ was affected by time-of-day (Figures 1C,D). The ratio between SLA and T_b_ indicates the relative importance of the locomotor activity levels to the daily changes in T_b_ (Machado et al., 2015). Higher ratios would represent the insensitivity of T_b_ to SLA, whereas lower ratios might suggest increased sensitivity to SLA (Figure 1C). Notably, we observed that this ratio exhibits a time-of-day-dependent oscillatory pattern (*F* = 11.81; *p* < 0.001; *n* = 23). This ratio shows a peak during the beginning of the dark-phase. In line with this finding, we also observed that the two parameters exhibited an oscillatory pattern in their regression line slopes for each time-of-day (Figure 1D). These slopes strengthen the assumption that greater locomotor activity during the dark-phase might be necessary to change body temperature. During the light-phase, T_b_ is more sensitive to slight changes in SLA.

### Influence of time-of-day on exercise and thermoregulatory capacity

**Figure 2:**
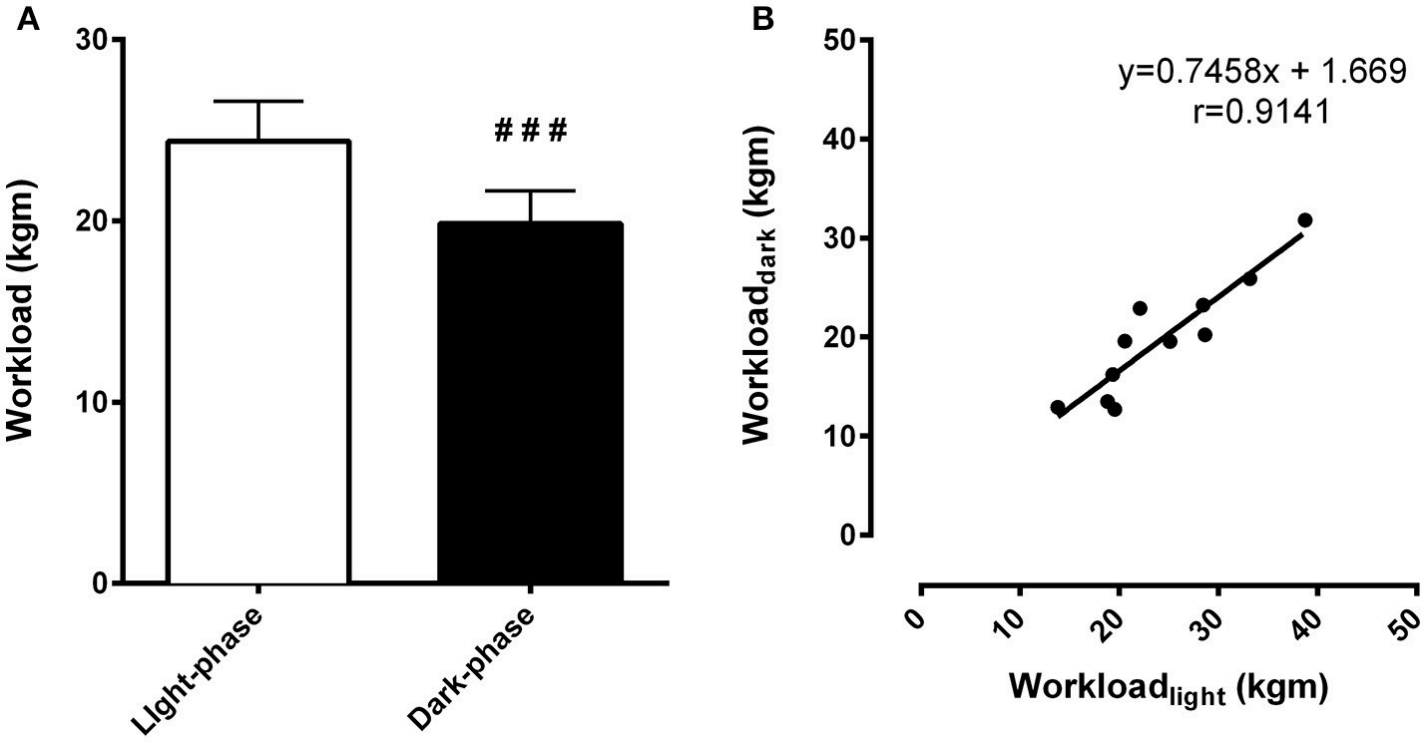
**Differences in exercise capacity during the light and dark-phases are independent of the variability of individual performance**. In **(A)**, total workload (kgm) is an index of exercise capacity. In **(B)**, correlation analysis between light and dark-phase workload calculated for each animal (*n* = 11) was performed to identify whether differences in exercise capacity could be related to the individual chronotype. Pearson's *r* coefficient was 0.9141 and *p* < 0.0001. ^*###*^Indicates *p* < 0.001 between phases. The results are expressed as the mean ± SEM (*n* = 11).

Exercise tests were performed during the light-phase and the dark-phase. During the light-phase, the time to fatigue was 15% higher compared to the dark-phase (46.53 ± 2.29 min vs. 40.50 ± 2.29 min, respectively, *p* < 0.01). The workload, an *index* of physical capacity adjusted by body weight, was 23% higher during the light-phase exercise compared to the dark-phase (24.39 ± 2.19 kgm vs. 19.86 ± 1.79 kgm, respectively, *p* < 0.001) (Figure 2A). This difference originates from time-of-day effects rather than individual variability as inferred by the correlational analysis between exercise performance during the light and dark-phases of the daily cycle. In fact, higher workload during one phase corresponded to increased exercise capacity during the other phase (*r* = 0.91, *p* < 0.0001) (Figure 2B).

**Figure 3:**
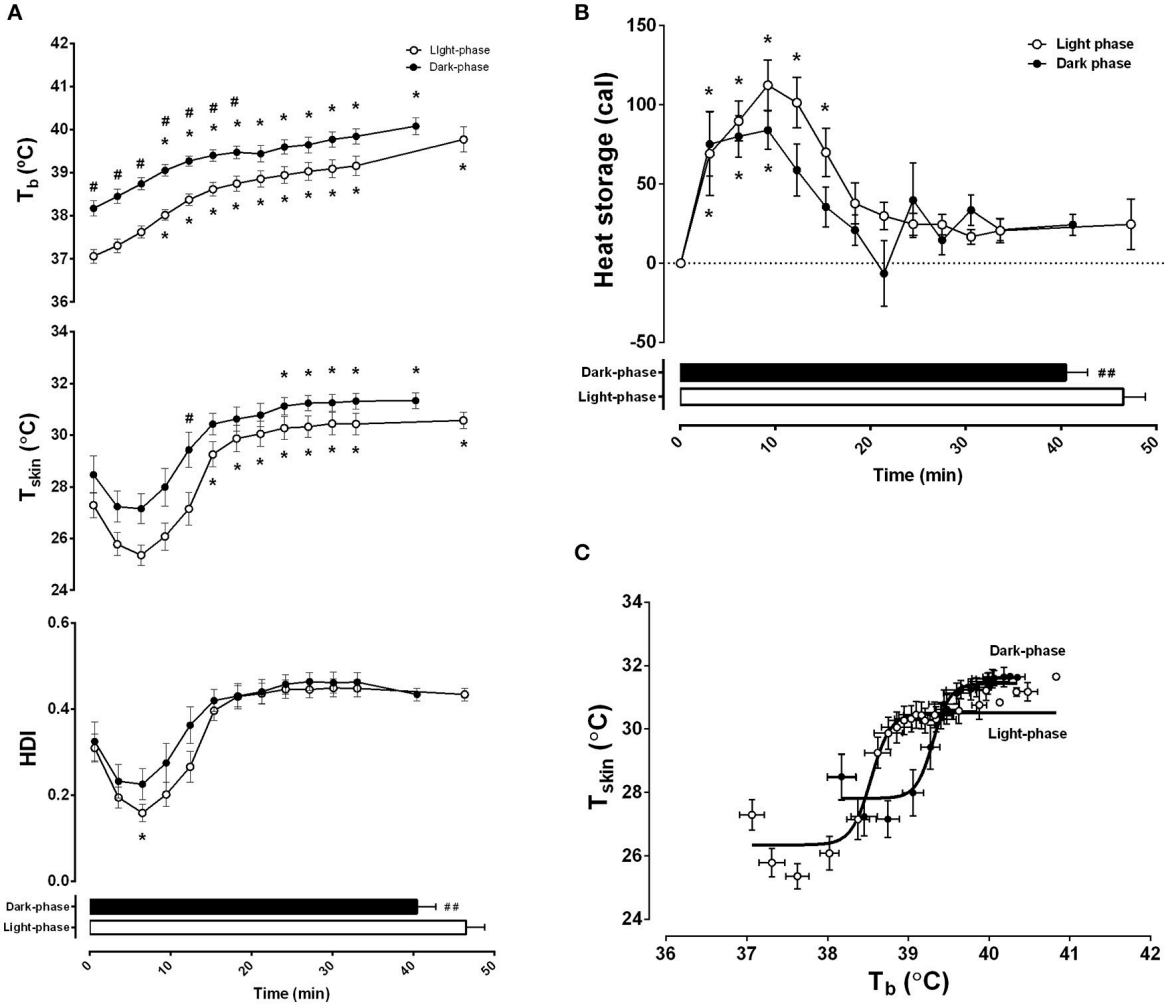
**Differences in exercise capacity during the light and dark-phases are associated with changes in thermal balance**. Light and dark-phase were represented as white and dark circles, respectively. In **(A)**, body temperature (T_b_ in °C), skin tail temperature (T_sk_ in °C), and heat dissipation index (HDI) are plotted as a function of exercise duration and performance (horizontal columns). In **(B)**, 3 min intervals of calculated heat storage are also plotted as a function of exercise duration. In **(C)**, a nonlinear four-parameter analysis of body temperature and skin temperature is presented as a representation of thermoregulatory effector activity. Best fit curves for light (*r* = 0.945, *p* < 0001) and dark (*r* = 0.933, *p* < 0.001) are presented as solid lines. The results are expressed as the mean ± SEM (*n* = 11). *Indicates *p* < 0.05 against 0 min of exercise; ^#^*p* < 0.05 and ^*##*^*p* < 0.01 between light and dark.

Thermoregulatory adjustments induced by graded exercise were also evaluated (Figure 3). Consistent differences were observed between exercise during the night and day. Progressive exercise induced an increase in T_b_ that was affected by time-of-day (*F* = 168.20, *p* < 0.0001) and exercise duration (*F* = 33.52, *p* < 0.0001) (Figure 3A, *top panel*). During the light-phase, T_b_ remained lower than during the dark-phase through the entire exercise duration until fatigue (39.8 ± 0.3°C vs. 40.1 ± 0.2°C, respectively). Because T_b_ is directly dependent on changes in heat storage and balance between the onset and the end of exercise, we tested whether the differences in the initial T_b_ observed at the two moments of the day influenced workload. Interestingly, only T_b_ at the onset of exercise during the light-phase was negatively correlated to workload (*r* = −0.61, *p* < 0.05).

Furthermore, both time-of-day and exercise duration affected T_sk_ (*F* = 37.83, *p* < 0.0001 and *F* = 24.46, *p* < 0.0001, respectively) (Figure 3A, *middle panel*) and HDI (*F* = 6.49, *p* < 0.05 and *F* = 21.76, *p* < 0.05) (Figure 3A, *bottom panel*). Maximal vasoconstriction, which corresponded to the lowest level of T_sk_, was observed at the 6th min of exercise in both conditions (25.4 ± 0.4°C during the light-phase and 27.2 ± 0.6°C during the dark-phase), although only during the light-phase was it different from the beginning of exercise. This was confirmed with the calculated heat dissipation index (HDI). The steady state skin vasodilation (corresponding to the moment when T_sk_ remained stable until the interruption of exercise) was reached after the 15th min of the light-phase (30.6 ± 0.3°C) and the 24th min of the night phase (31.1 ± 0.3°C). However, this change was not captured with the HDI analysis, indicating that both conditions reached a steady state after approximately 15 min of exercise. In addition, although the absolute T_sk_ at fatigue was higher during the dark-phase, the HDI was not different between the day and night experiments (*p* < 0.05).

The difference between the onset of skin vasodilation and heat production induced by physical activity results in heat storage and consequently, in increased T_b_. Interestingly, heat storage was affected by the duration of exercise and time-of-day (*F* = 32.72, *p* < 0.0001 and *F* = 1.15, *p* < 0.05, respectively) (Figure 3B). Although it remained augmented during the first 15 min of exercise during the light-phase, the increased heat storage effect lasted 9 min in the dark-phase. After that, heat storage remained steady until the end of the protocol. This was confirmed by a positive correlation between workload and total heat storage (*r* = 0.64, *p* < 0.01). Interestingly, this association was also dependent on time-of-day as the correlation was significant during the light-phase (*r* = 0.74, *p* < 0.01), but not the dark-phase (*r* = 0.34, *p* = 0.30).

Using a different analytical approach, i.e., effector analysis of T_b_ regulation through T_sk_ activity (Figure 3C), we observed that the sensitivity of the thermoregulatory system (represented by a sigmoidal best-fit curve) shifts to the right and upwards, thus limiting the rise in T_b_ during the dark-phase (*F* = 100.67, *p* < 0.0001). In line with our previous findings, the curve analysis demonstrated that the absolute threshold for vasodilation, calculated with LogEC50, was higher in the dark-phase (39.3 ± 0.02°C vs. 38.5 ± 0.02°C in the light-phase) without changes in slope/sensitivity (4.10 ± 0.58 vs. 3.57 ± 0.48 in the dark and light-phase, respectively).

### Influence of time-of-day on exercise-induced neuronal activation

**Figure 4:**
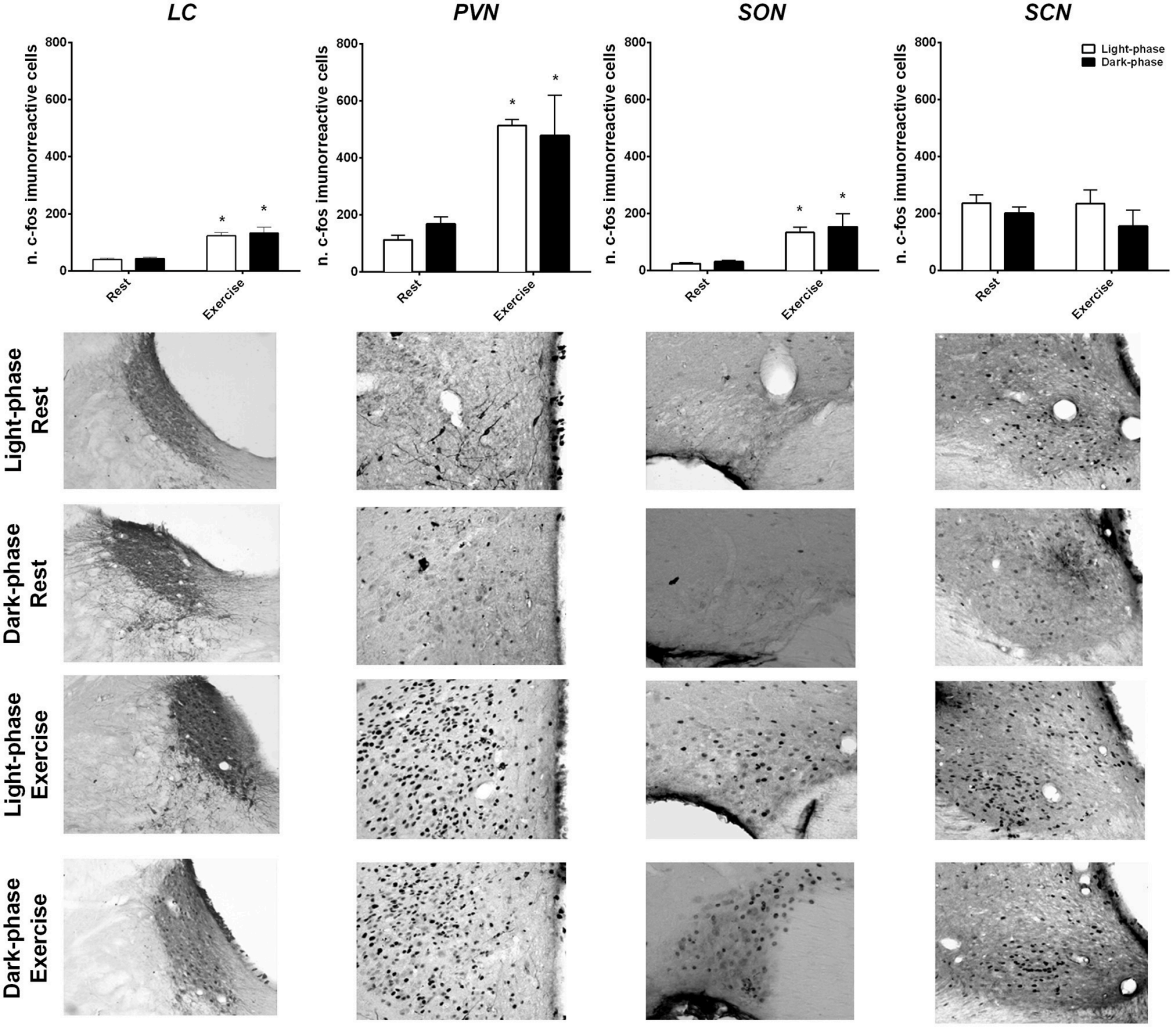
**Exercise-induced neuronal activation in the hypothalamus and the brainstem independent of time-of-day**. The number of c-fos immunoreactive proteins were quantified and graphically represented after rest and high-intensity exercise conditions during the light (white columns) and dark-phases (black columns). Representative photomicrographs of coronal brain sections (20X) of each nuclei are presented for each condition. The results are expressed as the mean ± SEM (*n* = 4–6/group). *Indicates *p* < 0.05 between rest and exercise.

Neuronal activation induced by exercise and time-of-day was evaluated by c-fos staining in the SCN, PVN, SON and LC (Figure 4). Exercise to fatigue activated the PVN (*F* = 20, *p* < 0.001), LC (*F* = 56.9, *p* < 0.001) and SON (*F* = 12.63, *p* < 0.001) regardless of time-of-day. Notably, the SCN was not affected by exercise, suggesting a resilience of this area to exercise load.

## Discussion

The most important finding of the present work was that exercise-induced neuronal activation was independent of time-of-day in brain regions related to homeostatic control, such as the PVN, SON and LC. In addition, we showed that the central clock in the SCN is resilient to high-intensity exercise. The present study also shows that time-of-day affected exercise capacity through changes in the heat storage dynamics and thresholds for tail vasodilation, without remarkable changes in thermal sensitivity. Taken together, our results points toward a role of the circadian system in the control of basal activity of the thermoregulatory system, especially at the onset of physical activity/exercise. The SCN modulation of basal activity in areas more directly involved in thermal balance, such as the medial and median pre-optic areas (MPOA and MnPO, respectively), or areas related to arousal, i.e., the lateral hypothalamus (LH), might be more suitable areas to explain how the CNS copes with the external load imposed by exhaustive activity.

Interestingly, we observed that the graded exercise test did not affect the SCN probably due to the resilience of the central clock when confronted with acute and chronic non-photic stimuli. In fact, in a rat model of simulated shiftwork, uncoupled daily neuronal PER1 activity between the SCN and other hypothalamic nuclei without changes in pacemaker phase was observed (Salgado-Delgado et al., 2010). Similarly, Per2::luc transgenic mice subjected to regular physical exercise during the light-phase changed the phase of PER2 in peripheral tissue but not in the SCN (Wolff and Esser, 2012). Temperature cycles within physiological ranges (36–38.5°C) also induced phase changes in the periphery independent of central pacemaker activity (Buhr et al., 2010). Therefore, it could be hypothesized that if exercise and changes in T_b_ affect the circadian timing system, these should be related to changes in CNS activity outside the central pacemaker and at other organs of the body. In line with this hypothesis, feeding is thought to synchronize the circadian system through changes in the molecular clock of the DMH, which is relatively independent of the SCN (Stephan et al., 1979; Stephan, 2002; Saper, 2013). Hence, the possibility that other *zeitgebers* might exert their actions through other brain nuclei remains to be further elucidated.

The daily cycles of light and dark synchronize endogenous circadian rhythms, such as body temperature and physical activity rhythms. Currently, it is accepted that the hypothalamic SCN directly or indirectly mediates this coupling between endogenous oscillations and external cues, at least in mammals. The SCN projects to other hypothalamic nuclei, such as the MPOA, subPVN, DMH and LH, to change their autonomic tone throughout a 24 h cycle (Kalsbeek et al., 2012). This autonomic modulation is implied in the control of daily energetic balance (Atkinson et al., 2007; Kotz et al., 2008) and could therefore be related to changes in the time-of-day exercise capacity that have been previously reported (Machado et al., 2015).

Our results showed that the control mechanisms involved in the daily changes of T_b_ follow a pattern of increased sensitivity during the light-phase and reduced sensitivity during the dark-phase, thus providing further evidence that circadian rhythms could be implicated in the modulation of the thermal state of the animal during the day (Weinert and Waterhouse, 1998; Waterhouse et al., 2005). During the active phase, the mechanisms of T_b_ control should be less affected by heat production derived from physical activity, which is likely due to the aroused state. During inactivity, this relationship might be inverted, leading to an increased influence of physical activity in T_b_ (Figures 1C,D). These results are in line with our findings from performance tests and subsequent thermoregulatory adjustments induced by exercise during the light and dark-phases. Time-of-day influenced exercise in a manner that, during the light-phase, the lower arousal state implicated lower initial T_b_ and increased heat storage capacity, both of which correlated with higher maximal physical capacity. These results confirmed our previous results (Machado et al., 2015) and further elucidated how the thermoregulatory adjustments induced by exercise could be affected by time-of-day.

Heat dissipation dynamics, measured through T_sk_, were not affected by time-of-day. However, the thresholds for vasodilation were higher during the dark-phase (Figure 3C). Notably, there were no differences between the moments when animals reached maximal vasoconstriction at the beginning of the activity and when they reached maximal vasodilation (Figure 3A). In contrast, heat storage seemed to be more affected by time-of-day and was consequently more influential on physical capacity (Figure 3B). Our results are in accordance with previous findings showing that rats running on a treadmill for 30 min at a low-to-moderate speed (12.5 m min^−1^) during the light or dark-phase showed only a small adjustment in thermal balance (Tanaka et al., 1990). Taken together with our findings, these results support the hypothesis that time-of-day differences in exercise performance and thermal balance during rest and exercise rely on the initial state of the exercising subject. This difference could lead to changes in autonomic balance during the onset of physical effort during the two phases of the daily cycle. In fact, our results showing the 24 h pattern of SLA/T_b_ and their slopes favor an oscillatory set point for T_b_. Moreover, it seems that the circadian system controls the basal activity of the thermoregulatory system and represents an important component of the onset of physical activity.

The importance of the initial state of thermal balance has been the topic of discussion regarding the physiological determinants of exercise performance (Webb, 1995; Briese, 1998; Gleeson, 1998; Coimbra et al., 2012). The traditional concept of a critical internal temperature (Fuller et al., 1998; Walters et al., 2000) has been more recently challenged by the idea that the rate of heat storage/dissipation could also play a role in exercise interruption (Rodrigues et al., 2003; Noakes et al., 2004; Lambert et al., 2005). Therefore, if heat storage during exercise is a function of the difference between the initial and final body temperature, it could be expected that time-of-day influences this process by changing the basal tone of the thermoregulatory system. Our results showing that heat storage was higher during the light-phase of the cycle and that it, as well as initial body temperature, was correlated with physical capacity strongly reinforces this concept. Furthermore, time-of-day affected the threshold for vasodilation during exercise without changing the slope and maximal body temperature. This suggests that the effect of the light or dark-phases on exercise performance occur at the level of activity thresholds for each effector of the thermoregulatory system rather than a direct CNS modulation of the refined adjustments induced by exercise. Remarkably, the manipulation of CNS neurotransmitters during exercise, rather than changing the limits of body temperature during exercise, modifies heat storage rates and dissipation during exercise (Soares et al., 2004; Lacerda et al., 2005; Meeusen et al., 2007; Pires et al., 2007; Balthazar et al., 2009, 2010; Leite et al., 2013; Lima et al., 2014a).

In alignment with this hypothesis, we investigated whether brain areas involved in the regulation of thermal balance and arousal were distinctly activated after exercise to fatigue in the light and dark-phases. Interestingly, regardless of time-of-day, the PVN, SON, and LC were intensely activated by exercise. These regions are implicated in the autonomic response to a stressful stimulus and are also activated by other stressor types, such as high intensity exercise (Pacák and Palkovits, 2001; Mastorakos et al., 2005; Soya et al., 2007; Yanagita et al., 2007; Barna et al., 2012). This activation seems to be dependent on exercise intensity and has been associated with lactate and glucose plasma levels (Soya et al., 2007). Taken together with our data, these results corroborate the concept of a complex central system that controls exercise adjustments and performance. However, time-of-day did not seem to affect the activity of the investigated brain areas, reinforcing the idea that the effect of light, and dark-phases modulates thermal balance at the effector level.

The thermoregulatory system is thought to be one of many factors that determine fatigue and exercise capacity (Cheung and Sleivert, 2004) and is regulated through parallel pathways of the CNS (Romanovsky, 2007; Morrison and Nakamura, 2011). The PVN is a major autonomic center for sympathetic activity, and its activation might be implicated in vasodilation and substrate mobilization in response to hyperthermia (Nagashima et al., 2000; Soya et al., 2007). This might explain why PVN activation was similar during the light and dark-phases. In fact, both T_b_ and HDI at fatigue during light and dark-phase exercise were similar. In previous reports it was observed that exercise to fatigue after a submaximal test (~66% VO_2_ max) induced PVN neuronal activation and that this effect was related to vasodilation capacity (Lima et al., 2014a). Furthermore, ACTH release, lactate accumulation, increased glycaemia, and adrenaline secretion were all associated with the level of PVN activation after exercise (Soya et al., 2007). Because the PVN is part of an axis that comprises the LC at the brainstem level, it is possible that LC activation might reflect this hypothalamic response to high intensity exercise integrated at the PVN.

In addition, the resting neuronal activity in the studied brain areas was not different at the two moments investigated. This might be explained by the timing of the measurements. In fact, the neuronal activation of these groups was measured 2 h after phase-change, ZT2 (light) and ZT16 (dark) representing two distinct orientations of activity although with the same level at those moments. This is in agreement with previous observations that the highest level of c-fos protein in the SCN is found during the late night while little difference (if any) is observed between ZT2 and ZT16 (Edelstein et al., 2000). Furthermore, previous work (Soya et al., 2007; Lima et al., 2014a; Santiago et al., 2016) showed that c-fos neuronal activation can detect strong changes in workload (~40–60%), however the present study showed a significant but less pronounced change (~20%). This might reflect a limitation of immunohistochemistry sensitivity that impairs the detection capacity for dynamic processes. Therefore, it is rather possible that SCN modulates basal activity and further influences physical activity onset.

## Conclusion

The present study showed that under normal laboratory conditions of a light/dark cycle and constant ambient temperature, exercise-induced neuronal activation was independent of time-of-day in brain regions related to homeostatic control, such as the PVN, SON and LC. In addition, we showed that the central clock is resilient to high-intensity exercise. The present study also shows that time-of-day affected exercise capacity through changes in the heat storage dynamics and thresholds for tail vasodilation, without remarkable changes in thermal sensitivity. During non-exercise conditions, body temperature is more responsive to physical activity. On the other hand, during exercise conditions, the absolute threshold for the increased activity of tail skin vasodilation considerably changed during each time-of-day. This resulted in higher workload and, consequently, increased heat storage capacity and in the light-phase, reinforcing the importance of the initial physiological status to reach maximal effort during exercise.

## Author contributions

FM and CC designed the experiment; FM and DF acquired the data; FM, DF, MP, and CC contributed to the analysis and interpretation of data; FM, DF, MP, and CC participated in the elaboration of the manuscript and gave the final approval for submission and publication, being accountable for all aspects of the present work.

### Conflict of interest statement

The authors declare that the research was conducted in the absence of any commercial or financial relationships that could be construed as a potential conflict of interest.
